# Enhanced Dibutyl Phthalate Sensing Performance of a Quartz Crystal Microbalance Coated with Au-Decorated ZnO Porous Microspheres

**DOI:** 10.3390/s150921153

**Published:** 2015-08-27

**Authors:** Kaihuan Zhang, Guokang Fan, Ruifen Hu, Guang Li

**Affiliations:** 1 State Key Laboratory of Industrial Control Technology, Institute of Cyber Systems and Control, Zhejiang University, Hangzhou 310027, China; E-Mails: zhangkaihuan@zju.edu.cn (K.Z.); guangli@zju.edu.cn (G.L.); 2 School of Chemistry and Chemical Engineering, Yangzhou University, Yangzhou 225002, China; E-Mail: gkfan@yzu.edu.cn

**Keywords:** ZnO porous microspheres, Au-decorated, dibutyl phthalate, gas sensor, quartz crystal microbalance (QCM)

## Abstract

Noble metals addition on nanostructured metal oxides is an attractive way to enhance gas sensing properties. Herein, hierarchical zinc oxide (ZnO) porous microspheres decorated with cubic gold particles (Au particles) were synthesized using a facile hydrothermal method. The as-prepared Au-decorated ZnO was then utilized as the sensing film of a gas sensor based on a quartz crystal microbalance (QCM). This fabricated sensor was applied to detect dibutyl phthalate (DBP), which is a widely used plasticizer, and its coating load was optimized. When tested at room temperature, the sensor exhibited a high sensitivity of 38.10 Hz/ppb to DBP in a low concentration range from 2 ppb to 30 ppb and the calculated theoretical detection limit is below 1 ppb. It maintains good repeatability as well as long-term stability. Compared with the undecorated ZnO based QCM, the Au-decorated one achieved a 1.62-time enhancement in sensitivity to DBP, and the selectivity was also improved. According to the experimental results, Au-functionalized ZnO porous microspheres displayed superior sensing performance towards DBP, indicating its potential use in monitoring plasticizers in the gaseous state. Moreover, Au decoration of porous metal oxide nanostructures is proved to be an effective approach for enhancing the gas sensing properties and the corresponding mechanism was investigated.

## 1. Introduction

Phthalates are multifunctional chemicals that have been widely used for decades and thus are widely found in the environment and in humans. In particular, as a kind of lower molecular weight phthalate, dibutyl phthalate (DBP) is employed in diverse applications, including solvents, adhesives, inks, pharmaceutical and cosmetic products, medical devices and materials made of PVC [1,2]. However, its ubiquitous presence also brings more risks. Animal studies have suggested that DBP not only affects the liver and kidneys, but also presents powerful reproductive and developmental toxicity effects and disrupts the endocrine system [3]. As a result of this potential estrogenic effect, DBP has been classified as an endocrine disrupting compound (EDC) [4], and a growing number of associated studies have been performed recently, even on humans, to assess the adverse effects of DBP exposure and to determine its concentration [5,6,7,8]. What’s more, as a semi-volatile organic compound (SVOC), DBP always exists as a low-concentration vapor besides its liquid-state exposure [2,9] and has been detected in various environments such as indoor air and dust [1,4,10,11,12], airborne particulate matter (PM) [13,14] and the air inside vehicles [15]. Considering the issues described above, there is an urgent need to detect DBP vapor in the environment. The main methods currently used are gas chromatography (GC) and gas chromatography-mass spectrometry (GC-MS), and the concentrations detected (median values) in the literature [1,4,10] are respectively 227 ng/m^3^, 220 ng/m^3^ and 1188 ng/m^3^. Although these techniques provide for low-level detection of phthalates, they always take considerable time and involve high instrumentation costs.

To obtain a rapid, easy and high sensitive vapor detection, quartz crystal microbalance (QCM) sensors have been a popular choice. By using appropriate sensitive coatings, a QCM is able to work at room temperature as a mass sensitive sensor and can maintain good sensitivity and high stability [16]. The Sauerbrey equation [17] clearly expresses the primary QCM operation principle for conversion of frequency to mass:

(1) $$\Delta f=\;-\;\frac{2\;f_{0}^{2}}{A\;\sqrt{\mu_{q}\rho_{q}}}\;\Delta m\;$$

where *∆f* is the observed frequency shift (Hz), *f_0_* is the fundamental frequency of the quartz crystal (MHz), *A* is the active area of the electrode (cm^2^), *ρ_q_* is the density of quartz (2.649 g/cm^3^), *μ_q_* is the shear modulus of quartz (2.947 × 10^11^ g/cm/s^2^) and *∆m* is the mass change on the active surface of the crystal (g). This equation is only applicable when the deposited mass has the same acoustic properties as the crystal and the frequency change is small and may be affected by various factors. When the application conditions change, corresponding modifications to the equation are needed [18]. Based on this principle, nanogram-level mass changes can be determined by measuring the frequency shift (*Δf*) caused by the adsorption of target analytes onto the coated-QCM. The sensitive coatings range from polymers [19,20] to metal oxides [21,22], and even to other functional materials [23,24]. In order to further improve the gas sensing properties of QCM sensors, nanostructured hybrid materials have received increasing attention in recent years because these materials are synthesized by composition, doping, decoration and incorporation and they always combine the advantages of all components and possess better sensing performance [25,26,27,28]. 

In this paper, the synthesis of nanostructured zinc oxide (ZnO), its surface modification and gas sensing application were investigated. ZnO is a typical n-type semiconductor which displays distinctive electrical, optical and electrochemical properties and has various applications such as transparent electrodes [29], solar cells [30], photocatalysts [31] and gas sensors [32,33]. Although it is abundant in nature and low-cost, it retains high sensitivity, excellent stability, non-toxicity and suitability for doping, especially for gas sensing applications [34]. Numerous literatures have reported its utilization as a gas sensor. Recent associated studies are mainly focused on controlling the growth of nanomaterials with a designed size, shape, and morphology [35,36], and on the surface modification with noble metals or other materials [37,38,39,40]. Based on a previous study [41], hierarchical ZnO porous microspheres were successfully synthesized. These hierarchical ZnO microspheres were formed by self-assembling nanosheets and possessed a large specific surface area. Then Au particles were decorated on the surface of the porous microspheres to enhance the gas sensing properties attributed to the high catalytic activity of noble metals towards test gases. The detailed characterization and performance of this material were reported in our previous work [39].

In this study, the obtained Au-decorated ZnO was used as the sensing film to fabricate a highly sensitive QCM for DBP detection at room temperature. To our knowledge, there are few reports on such a unique system that combines Au-functionalized ZnO porous microspheres with a DBP sensor based on QCM. For the fabricated sensor, the coating load of the sensing material was optimized and sensor performance including sensitivity, selectivity and long-term stability were evaluated. According to the experimental results, the ZnO microspheres displayed great improvements both in sensitivity and in selectivity after Au decoration. The corresponding mechanisms responsible for the high sensitivity, selectivity and the enhancement caused by Au decoration were also investigated.

## 2. Experimental Section

### 2.1. Materials

The chemicals and reagents for preparing the sensing materials: zinc acetate dehydrate [Zn(CH_3_COO)·2H_2_O], hexamethylenetetramine (C_6_H_12_N_4_), sodium citrate (C_6_H_5_Na_3_O_7_·2H_2_O) and chloroauric acid hydrate (HAuCl_4_·4H_2_O), were purchased from Sinopharm Chemical Reagent Co., Ltd. (Shanghai, China). They were all analytical grade and used as received without further purification. The chemicals for preparing the analytes—dibutyl phthalate (DBP), diethyl phthalate (DEP), dimethyl phthalate (DMP), ethanol, toluene, acetone, *n*-hexane, trichloromethane (chloroform) and ethyl acetate were purchased from Sigma-Aldrich (Shanghai, China). Deionized water and anhydrous alcohol were used throughout the experiments. The AT-cut 6.0 MHz (HC-49/U) quartz crystals were purchased from Kesheng Electronics Ltd. (Yantai, China). 

### 2.2. Preparation of ZnO Porous Microspheres and Au-Decorated ZnO Porous Microspheres

#### 2.2.1. Synthesis of ZnO Porous Microspheres

The synthesis process of the hierarchical porous ZnO microspheres was similar to that reported in the literature [39,41]. Zn(CH_3_COO)·2H_2_O (0.01 mol), C_6_H_12_N_4_ (0.01 mol) and C_6_H_5_Na_3_O_7_·2H_2_O (0.001 mol) were dissolved in deionized water (100 mL). After stirring for 30 min, the mixed solution was kept at 95 °C for 4 h. When the reaction completed, the white precipitates in the solution were collected and washed several times. Then the product was annealed at 400 °C for 30 min and the ZnO porous microspheres were obtained finally.

#### 2.2.2. Preparation of Au-Decorated ZnO Porous Microspheres

The ZnO porous microspheres were decorated with Au particles according to the method reported in [42] with some modifications. As-prepared ZnO porous microspheres (40 mg) were dispersed in deionized water (100 mL) and HAuCl_4_ (0.24 M, 0.11 mL) was added. The solution was heated at 110 °C under stirring for 15 min and then 0.04 M C_6_H_5_Na_3_O_7_ solution (3 mL) was added to reduce the HAuCl_4_ to metallic Au particles. A pink precipitate was obtained by centrifugation after stirring for 40 min. Finally, the precipitate was washed with deionized water and anhydrous alcohol several times and dried in a vacuum stove at 60 °C for 12 h. Then the ZnO porous microspheres decorated with Au particles were obtained as a pink powder. The Au-free and Au-containing samples were named ZnO and ZnO-Au, respectively.

### 2.3. Characterization

The X-ray diffraction (XRD) analysis for phase identification was performed using a X-ray diffraction device (X’Pert PW3050/60, PANalytical, Nijmegen, The Netherlands) running with Cu Kα radiation at an angle degree range from 20° to 80° (2*θ*). The scanning electron microscopy (SEM) analysis for morphology and microstructure investigation was performed with a S4800 Field-Emission Scanning Electron Microscope (Hitachi, Tokyo, Japan).

### 2.4. Fabrication of the QCM Sensing Film

The quartz crystal used in our experiments was AT-cut 6.0 MHz with Ag-coated electrodes. The electrode area is 0.196 cm^2^ (with 5 mm diameter), and the resulted theoretical sensitivity based on Sauerbrey equation is 4.144 × 10^8^ Hz/g. First, the crystal was rinsed with deionized water and anhydrous alcohol using an ultrasonic cleaner and dried in high–purity N_2_ at room temperature. The as-prepared samples were dispersed homogeneously in deionized water and ultrasonicated to form a uniform sample mixture solution with concentration of 10 mg/mL. After standing for 24 h, the sample mixture was dispensed onto both sides of the electrode surface of QCM using drop-coating method via a high-precision micropipette. The QCM sensors coated with nanostructured films were then obtained following a 24-h drying process. 

### 2.5. Preparation of Measured Vapors

The vapors used for this investigation were DBP, ethanol, toluene, acetone, *n*-hexane, chloroform and ethyl acetate. Their concentrations were determined based on static headspace sampling method [43]. Small amounts of analyte solutions were injected into 1 L glass container via a micro syringe and were left to vaporize. Accordingly, the vapor concentrations were calculated according to the following gas law [44]:

(2) $$c=\;\frac{22.4\rho TV_{s}}{273MV}\;\times \;10^{3}$$

where *c* is the concentration (ppm), *ρ* is the density of the liquid sample (g/mL), *T* is the temperature of vapor container (Kelvin), *V_s_* is the volume of the liquid sample (µL), *M* is the molecular weight of analyte (g/mol), and *V* is the container volume (L). Since the vapor pressure of DBP at 25 °C is 2.01 × 10^−5^ mm Hg, it exists as a low–volatility liquid at room temperature [45]. The concentration of saturated DBP vapor at 25 °C was determined using a triple quadrupole GC-MS system (7000C, Agilent Technologies, Santa Clara, CA, USA) as 842 ppb and other concentrations of DBP were obtained by diluting the saturated vapor [21]. During all the processes, high-purity N_2_ was used as the diluent gas. 

### 2.6. Measurement of Gas Sensing Properties

The sensing properties of the sensors were evaluated by measuring the QCM frequency shifts caused by the additional mass loadings. The measurement device was consisted of a sensing film coated crystal and an uncoated crystal, which were used as the sensing QCM and the reference, respectively. Both the sensing QCM and the reference QCM were oscillated by a driving circuit. Accordingly, the frequency difference between the reference and sensing QCM was measured using a frequency counter and recorded as the sensor output by a personal computer via a RS-232 serial communication port every second. The sensitivity of this system is comprehensively determined by Sauerbrey equation, the application conditions as well as the related parameters of the driving circuit. The sensors were measured in a 1000 mL sealed chamber and all experimental procedures were carried out at room temperature (25 ± 1 °C) and atmospheric pressure (about 1 atm). Since the frequency of a QCM may be affected by pressure in the gas chamber, an elastic bag was connected to the chamber through a thin catheter to balance the pressure change caused by analyte injection. The whole measurement system was homemade and was described in detail in our previous work [21]. For each measurement, N_2_ was first used to purge the test chamber. When the baseline of the sensor was stable, certain volumes of prepared analytes were introduced into the chamber and the corresponding change of frequency difference was defined as the sensor response and was measured. Until a stable sensor response achieved, N_2_ was again used to desorb the sensor.

## 3. Results and Discussion

### 3.1. XRD Diffraction

**Figure 1:**
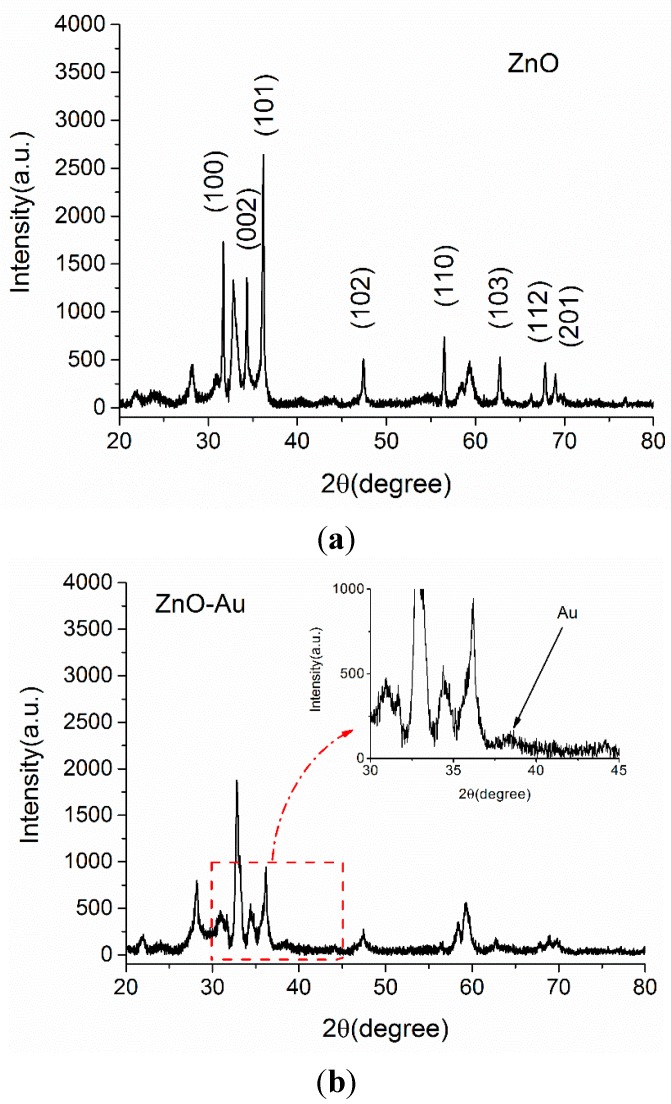
XRD patterns of the crystalline (**a**) ZnO and (**b**) Au-decorated ZnO. The inset of part (**b**) shows a magnification of the pattern at 2*θ* values ranging from 30° to 45° for Au-decorated ZnO.

The X-ray diffraction spectrum of pristine ZnO microspheres and those decorated with Au particles were shown in Figure 1a,b. As labeled in Figure 1a, the main feature peaks were at 31.8, 34.4, 36.3, 47.5, 56.6, 62.9, 67.9 and 69.1 degrees and were well matched with the standard data of the Zincite, syn (JCPDS card no. 36-1451), indicating that the sample was ZnO. However, there was a strong peak at 32.8° that couldn’t be determined. This undetermined peak indicated the existence of polycrystalline components resulted from the anisotropy of the condition in the synthesis process. In Figure 1b, an extra weak peak at 38.3° was observed besides the pure ZnO peaks, corresponding to the (111) planes of face-centered-cubic Au (JCPDS card no. 01-1174). From the XRD patterns, the crystalline structures of ZnO and ZnO-Au can be identified and the weak diffraction peak for Au implies the low content of Au in the product [46].

### 3.2. SEM Morphology

**Figure 2:**
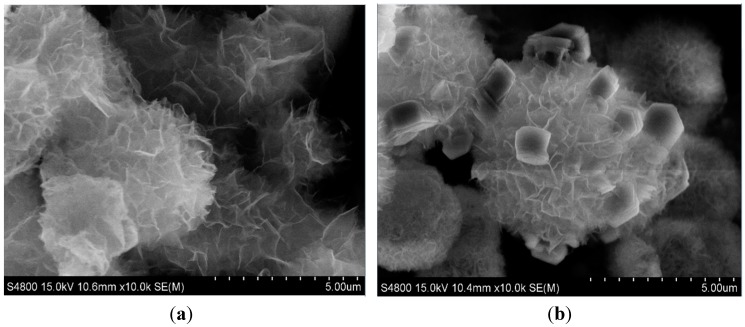
SEM images of the ZnO (**a**) and the Au-decorated ZnO (**b**) porous microspheres.

The morphologies of ZnO microspheres and those decorated with Au particles were examined by scanning electron microscopy (SEM) measurements and are shown in Figure 2. In both Figure 2a,b, hierarchical microspheres having a diameter of 3–5 µm can be observed. These hierarchical ZnO microspheres were formed by the self-assembling of nanosheets with a thickness of less than 50 nm. Compared with the SEM images in Figure 2a, cubic Au particles with a diameter from 500 nm to 800 nm are observed in Figure 2b. These Au particles were distributed on the surface of the inherited ZnO microspheres. 

### 3.3. Optimization of the Coating Load of Nano-Structured Sensing Materials

**Figure 3:**
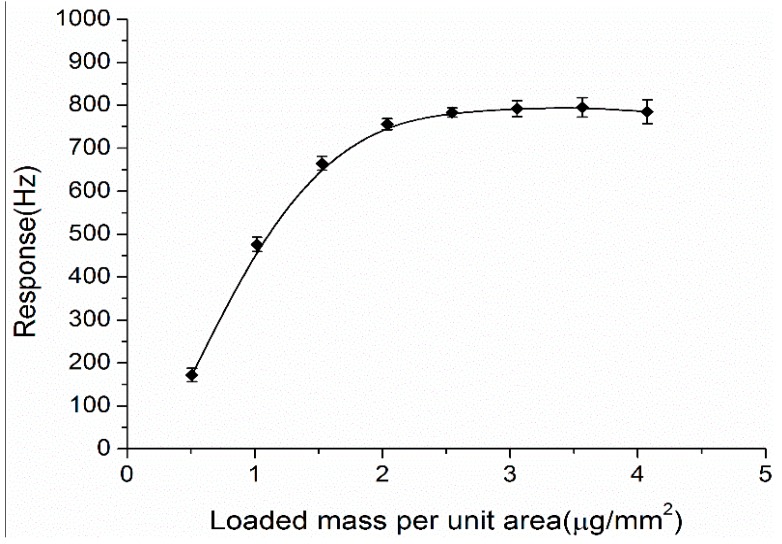
Responses of the Au-decorated ZnO coated QCM sensors with different coating load to 20 ppb of DBP.

Previous investigations confirmed that the coating load of the sensing material on the QCM sensors greatly affected both the mass-sensing properties and the adsorption of target analytes [32,47]. Thus, eight sensors with different deposited amounts of the prepared Au-decorated ZnO mixture were fabricated and tested to select the most suitable coating load for DBP detection. The coated mass per unit area (µg/mm^2^) was used to evaluate the coating load of the sensing film according to the principle of the QCM sensor. All the sensors were coated on both sides of the crystal electrodes uniformly and their responses to 20 ppb DBP were measured and are presented in Figure 3. The DBP response increased dramatically when the coating load increased from 0.51 to 2.04 µg/mm^2^. When the coating load was more than 2.04 µg/mm^2^, the responses did not increase significantly, however the response time and recovery time of the sensor became longer since the film thickness increased along with the coating load. To maintain both large response amplitude and fast response time, 3.06 µg/mm^2^ was selected as the optimal coating load and was adopted in all other experiments. 

### 3.4. Repeatable Response of the ZnO-Au Coated QCM Sensor to DBP

**Figure 4:**
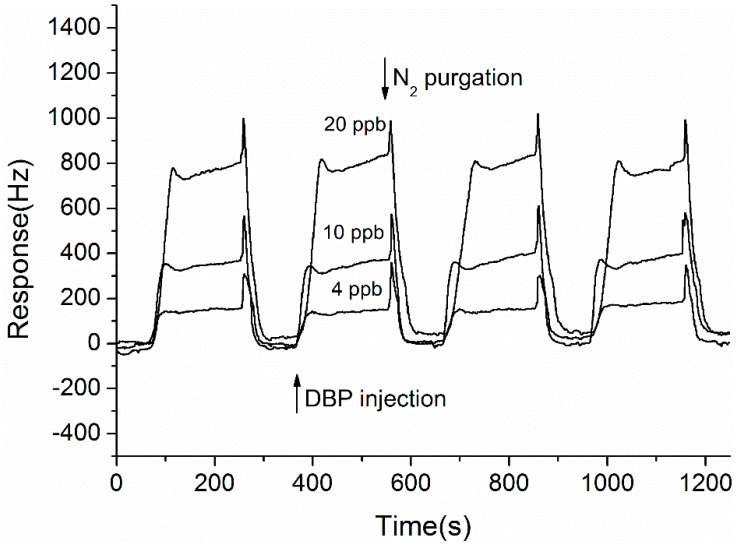
Response cycles of a ZnO-Au coated QCM sensor to 4, 10, and 20 ppb (from bottom to top) of DBP.

To evaluate the gas sensing performances, the optimized QCM sensor was repeatedly tested against different concentrations of DBP. For each measurement, four response cycles were run by alternatively exposing the sensor to 200-s analyte and 100-s N_2_. Figure 4 shows the dynamic response curves of the ZnO-Au QCM sensor to 4, 10 and 20 ppb of DBP. As can be seen, the sensor presented rapid and high responses to DBP, indicating the good sensing properties of the Au-decorated ZnO film. Moreover, the reversible dynamic response and the fast response and recovery time (t_90_, both less than 50 s) suggested that the DBP adsorption on the sensing film was mainly physical type caused by intermolecular force.

### 3.5. Sensitivity to Different Concentrations of DBP 

**Figure 5:**
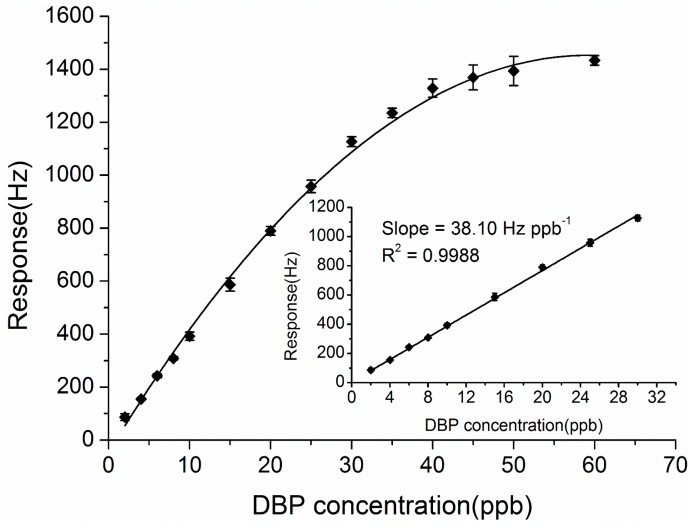
Calibration curve of the ZnO-Au QCM sensor response against the concentrations of DBP vapor. The inset shows the linear range of the sensor.

The responses of the ZnO-Au coated QCM sensor to DBP ranging from 2 ppb to 60 ppb were measured and are displayed in Figure 5. As seen from Figure 5, the sensor response increased along with the concentration of DBP and gradually reached a saturation level when the DBP concentration was above 30 ppb. In the low concentration range from 2 to 30 ppb (inset of Figure 5), an excellent linear relationship between the response of the sensor (*R*) and the DBP concentration (*C*) was obtained, as the Sauerbrey equation describes [17]. The regression equation is expressed as *R* = 38.10*C* + 5.78 with a correlation coefficient of 0.9988. The resulting sensitivity is 38.10 Hz/ppb, which represents a great improvement compared with our previous results (4.91 Hz/ppb, reported in [21]). The detection limit calculated as three times the signal-to-noise ratio was about 0.66 ppb.

**Figure 6:**
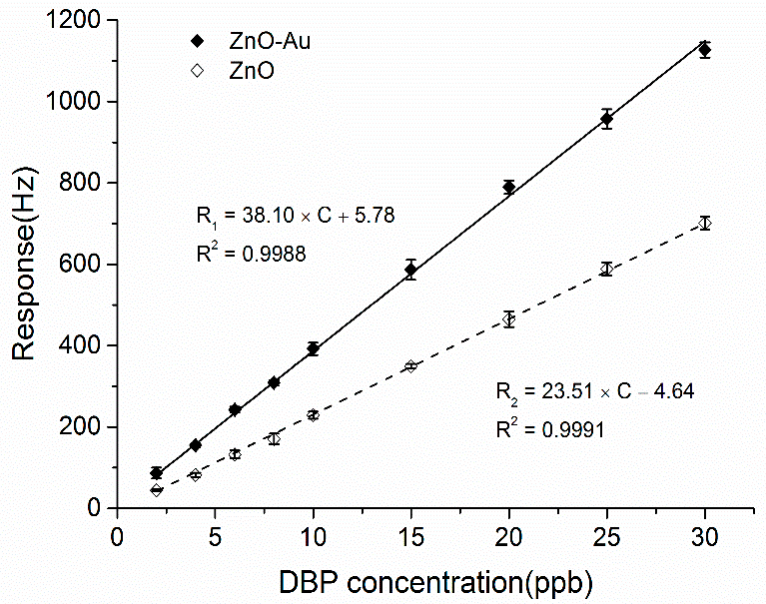
Sensitivity plots for the ZnO-Au and ZnO film coated sensors obtained from the dynamic tests; the lines are the linear fittings of the experimental data.

For comparison, the sensitivity of the ZnO coated QCM sensor was also tested and the results are plotted in Figure 6. The ZnO coated sensor also gave a response linearly proportional to the DBP concentration over the range from 2 to 30 ppb and achieved a sensitivity of 23.51 Hz/ppb. Both sensors were highly sensitive to low concentrations of DBP, which was mainly attributed to the hierarchical nanostructure of the ZnO porous microspheres. These porous microspheres provide a large specific surface area with more active sites and high surface accessibility. Moreover, the voids and interspaces among the interconnected nanosheets greatly facilitate the gas diffusion and transport in the sensing layers. The sensitivity of the ZnO-Au sensor which was functionalized with Au particles was enhanced 1.62 times compared with that of the pristine ZnO sensor because of the addition of catalytically active metals like Au, uniformly distributed and finely dispersed as catalytic clusters on the surface of the ZnO porous microspheres. The resulting catalytic activation and chemical sensitization can thus enhance the gas adsorption response [37,39]. Moreover, as reported in the literature [28,37,38], the Au content for decoration will greatly affect the sensing performance of the sensor. For this reason, further related research is currently underway to investigate how the amount and the size affect the sensing properties.

### 3.6. Selectivity of the ZnO-Au and ZnO Coated QCM Sensors

**Figure 7:**
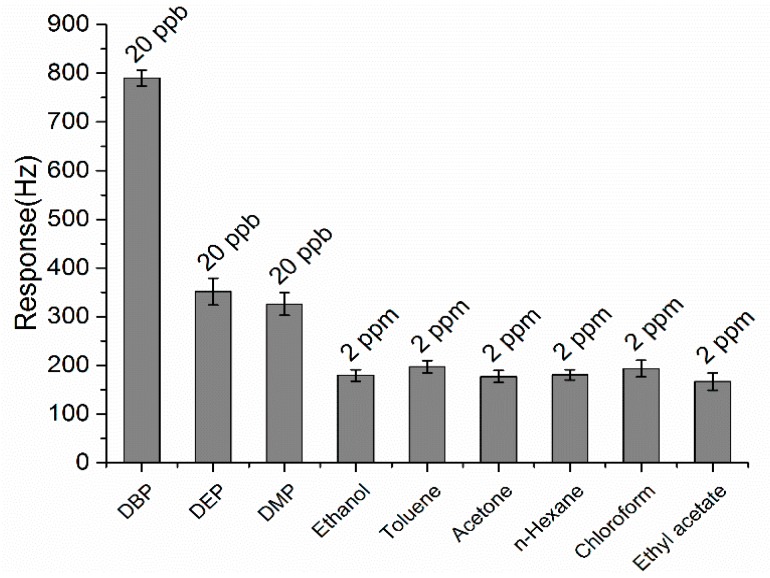
Comparison of the responses of the ZnO-Au QCM sensor to various organic vapors. DBP, DEP and DMP were measured at 20 ppb while the others were at 2 ppm.

The selectivity of the ZnO-Au QCM sensor was investigated by comparing the responses of the sensor to various volatile organic compounds (VOCs). Among them, DBP was analyzed as the target analyte and was measured at 20 ppb, while several conventional solvents like ethanol, toluene, acetone, *n*-hexane, chloroform and ethyl acetate acted as potential interferences and were tested in 2 ppm. Besides, two other phthalates, DEP and DMP, were also tested at 20 ppb. The corresponding response amplitudes are shown in Figure 7. Compared with the interferences caused by ethanol, toluene, acetone, *n*-hexane, chloroform and ethyl acetate, the response to DBP was about four times higher although the tested DBP concentration was only 1% of the others’. Therefore, the sensor possessed high selectivity to gaseous DBP against the six potential interferences. Meanwhile, the 20 ppb of DEP and DMP also caused significant responses, though lower than that of DBP. These results suggested that all three phthalates can be detected at ppb levels and indicated a superior affinity of the sensor to these phthalates. Since both DBP, DEP and DMP contain more hydrogen bond acceptors (4) than the others (≤2), they can more easily interact with the –OH groups on the surface of the nanostructured ZnO through hydrogen bonding and thus facilitate the physical adsorption. What’s more, the benzene ring and symmetrical molecular structure of the phthalates DBP, DEP and DMP lead to their good hydrophobicity, which contributes to their affinity and greatly enhances the adsorption capacity on the sensor. As a result, the sensor shows higher response to the phthalates over the other compounds [39]. Among DBP, DEP and DMP, the response amplitudes decreased in turn although they share similar structures and contain the same count of hydrogen bond acceptors. The length of the alkyl chains in the target analytes may account for this.

**Figure 8:**
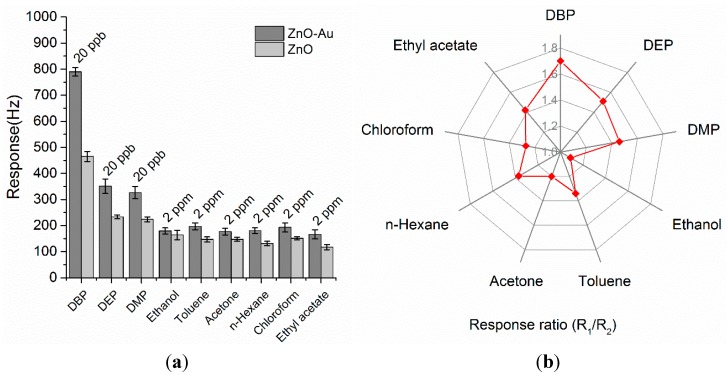
(**a**) Comparison of the responses of the ZnO-Au QCM to various organic vapors (R_1_) with that of the ZnO QCM (R_2_); (**b**) Polar plot of response ratio (R_1_/R_2_) between the ZnO-Au coated and the ZnO coated QCM.

In order to identify the role of Au-decoration on improving gas sensing properties, the pristine ZnO coated QCM was also exposed to the same VOCs and their responses were recorded as comparison. According to the results from Figure 8a, both sensors based on ZnO-Au and ZnO films were more sensitive to DBP vapor than to the other VOCs. Meanwhile, the Au-functionalized ZnO exhibited extraordinarily higher sensitivity than the pristine ZnO sensor, indicating that the gas sensing properties of the ZnO porous microspheres were apparently enhanced by Au particles decoration. In fact, as an n-type semiconductor, ZnO has both electronic and chemical sensitization [37,40,48] when Au particles were incorporated, which will greatly facilitate the formation of hydrogen bonds between the analyzed vapors and the sensitive film. Furthermore, Figure 8b shows the polar plot of response ratios (R_1_/R_2_, R_1_ and R_2_ represent the responses of the ZnO-Au QCM and ZnO QCM, respectively) to various vapors. The response ratios were all greater than one, but varied with the analytes. Interestingly, the experimental results indicated that DBP achieved a higher response ratio than the other vapors, implying an improvement of the selectivity to DBP. Besides, the response ratios of the three phthalates are more or less greater than that of the interferences. The richer hydrogen bond acceptors of DBP, DEP and DMP, their good hydrophobicity, together with the Au-sensitization are comprehensively responsible for this. However, the underlying mechanism for the sensitivity and selectivity enhancement by Au decoration still needs a deeper understanding.

### 3.7. Long-Term Stability

**Figure 9:**
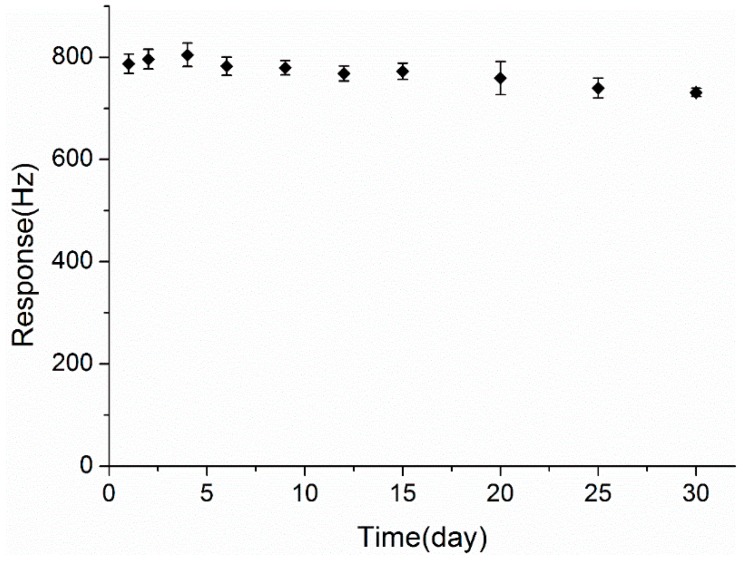
Long-term stability of the ZnO-Au QCM sensor to 20 ppb of DBP.

For practical applications, stability in long-term operation is required for gas sensors. Thus, the responses of the ZnO-Au QCM sensor to 20 ppb of DBP were regularly tested during 30 days to evaluate its gas sensing stability. During this period, the sensor was stored in a dry cabinet at room temperature after each test. As illustrated in Figure 9, the sensor response showed an acceptable change during the storage period and decreased only 7.11% from its original measured value after 30 days. This indicated that the sensor maintained good long-term stability.

## 4. Conclusions

Using a simple hydrothermal process followed by calcination, self-assembled hierarchical ZnO porous microspheres were synthesized. Then cubic Au particles were decorated on the surface of the ZnO porous microspheres via a facile aqueous solution method. Based on this Au-decorated ZnO film, a QCM sensor was developed to detect low-concentrations of DBP. At room temperature, the sensor was highly sensitive to DBP and achieved a ppb-level detection. Besides, the sensor also exhibited remarkable selectivity as well as acceptable long-term stability. These results strongly suggest that the hierarchical Au-decorated ZnO porous microspheres are a promising candidate for gas-sensing applications in monitoring harmful gaseous plasticizers. However, for practical applications, effective measures should be developed to prevent the interferences when used in real humid conditions. An undecorated ZnO based QCM sensor was also fabricated and evaluated in the similar way to determine the effects of Au decoration on gas sensing performance. The corresponding results indicated that both the sensitivity and selectivity of the sensor were significantly enhanced after Au decoration. This provides a potential way to improve the gas sensing properties of nanostructured metal oxides. In future study, relevant experiments will be designed to further explore the selectivity of the DBP sensor and to deeply investigate the mechanism of gas-sensing enhancement caused by Au decoration.
